# Proinflammatory cytokines tumor necrosis factor-α and interferon-γ modulate epithelial barrier function in Madin-Darby canine kidney cells through mitogen activated protein kinase signaling

**DOI:** 10.1186/1472-6793-6-2

**Published:** 2006-02-21

**Authors:** David M Patrick, Amanda K Leone, Jeffry J Shellenberger, Kara A Dudowicz, Jonathan M King

**Affiliations:** 1 Southwestern Graduate School of Biomedical Sciences, UT Southwestern Medical Center, 5323 Harry Hines Blvd., Dallas, TX 75390-9004, USA; 2 UT Medical School, UTHSCSA, 7703 Floyd Curl Drive, San Antonio, TX 78229-3900, USA; 3 Trinity University, Biology Department, One Trinity Place, San Antonio, TX 78212, USA

## Abstract

**Background:**

The tight junction is a dynamic structure that is regulated by a number of cellular signaling processes. Occludin, claudin-1, claudin-2 and claudin-3 are integral membrane proteins found in the tight junction of MDCK cells. These proteins are restricted to this region of the membrane by a complex array of intracellular proteins which are tethered to the cytoskeleton. Alteration of these tight junction protein complexes during pathological events leads to impaired epithelial barrier function that perturbs water and electrolyte homeostasis. We examined MDCK cell barrier function in response to challenge by the proinflammatory cytokines tumor necrosis factor-α (TNFα) and interferon-γ (IFNγ).

**Results:**

Exposure of MDCK cells to TNFα/IFNγ resulted in a marked sustained elevation of transepithelial electrical resistance (TER) as well as elevated paracellular permeability. We demonstrate that the combination of TNFα/IFNγ at doses used in this study do not significantly induce MDCK cell apoptosis. We observed significant alterations in occludin, claudin-1 and claudin-2 protein expression, junctional localization and substantial cytoskeletal reorganization. Pharmacological inhibition of ERK1/2 and p38 signaling blocked the deleterious effects of the proinflammatory cytokines on barrier function.

**Conclusion:**

These data strongly suggest that downstream effectors of MAP kinase signaling pathways mediate the TNFα/IFNγ-induced junctional reorganization that modulates MDCK cell barrier function.

## Background

Tight junction proteins combine to form an important barrier which serves to limit paracellular transport in epithelial cell lines [1]. Important studies have identified occludin [2], junctional adhesion molecule (JAM) [3] and the claudins [4] as tight junction proteins that restrict molecular movement within the paracellular space. These tight junction proteins form a dynamic seal between epithelial cells becoming the principle physical paracellular barrier. The extracellular domains of adjacent occludin or claudin molecules form interactions that restrict diffusion [5]. The claudin family, with now more than twenty members, has garnered much attention due to the heterogeneous expression patterns observed in a variety of epithelia and endothelial cell types. Complex arrays of claudin species with unique distribution patterns are found in each segment of the kidney [6]; for instance the distal tubule contained measurable expression of both claudin-3 and -8. MDCK II cells express claudin-1, -2, -3 and -4 [7], the expression of claudin-2 in the MDCK II cells is in part responsible for its low electrical resistance profile [8].

The response of the kidney epithelium to inflammatory mediators is complex; an early study demonstrated that Tumor Necrosis Factor-α(TNFα) exposure impaired barrier function [9]. The renal epithelium has been shown to produce TNFα as well as other potent proinflammatory cytokines in response to external stressors such as ischemia-reperfusion injury [10,11]. TNFα mRNA levels increased significantly thirty minutes after ischemia and inhibition of TNFα bioactivity decreases neutrophil infiltration and preserves renal function [12]. Investigation of the proximal tubule LLC-PK cell model shows that in order to produce a compromised epithelium the TNFα dose must elicit apoptosis [13,14]. The combination of TNFα and Interferon-γ (IFNγ) exposure in model epithelial cell lines such as Caco-2 cells [15,16] and T84 cells [17,18] results in loss of TER and increased paracellular permeability. Finally, in a recent MDCK cell study using a model of chronic exposure to TNFα for five days, claudin-1 expression eventually decreased and tight junctions were disrupted [19].

Mitogen-activated protein (MAP) kinases a family of serine-threonine kinases, have a fundamentally important roles as signal transducers. Activation of MAP kinases by various growth factors and cytokines are important molecules involved in modulating cellular responses [20,21]. In terms of tight junction regulation the role of MAP kinase signaling has been of interest [22,23]. MAPK kinase (MEK) overexpression led to epithelial dedifferentiation in MDCK-C7 cells [24]. Tight junction biogenesis was inhibited in MDCK cells expressing constitutively active MAP kinase; pharmacological inhibition of MEK1 signaling in these cells permitted tight junction formation [25]. Pharmacological inhibition of MEK, a Ras effector known to phosphorylate extracellular signal-regulated kinase 1 and 2 (ERK1 and ERK2), attenuated dexamethasone-induced tight junction formation in the Con8 mammary tumor cell line [26]. In these studies, the mitogenic effect of MAP kinase activity is logically opposed to tight junction formation. The analysis of the effects of external stimuli on tight junction regulation, specifically the activated signaling pathways, will provide valuable insight into tight junction regulation.

The goal of this present study was to characterize the response of MDCK cells to the combination of TNFα/IFNγ. We hypothesized that TNFα/IFNγ would impair MDCK cell tight junction function. We examined TER, paracellular flux, tight junction protein expression and localization in response to the proinflammatory cytokines. In a variety of disease states inflammation is thought to negatively impact epithelial barrier function, we report that TNFα/IFNγ co-administration to MDCK cell monolayers impaired epithelial barrier function as measured by elevated paracellular flux and produced marked elevation in transepithelial electrical resistance (TER). Occludin, claudin-1 and claudin-3 protein expression was induced by TNFα/IFNγ exposure, whereas claudin-2 levels decreased; tight junction protein localization was modulated contributing to impaired tight junction function. Inhibition of MEK1 and p38 signaling during exposure to TNFα/IFNγ, abrogated these cytokine-induced effects in MDCK cells.

## Results

### Effect of TNFα and IFNγ on cellular cytotoxicity

**Figure 1:**
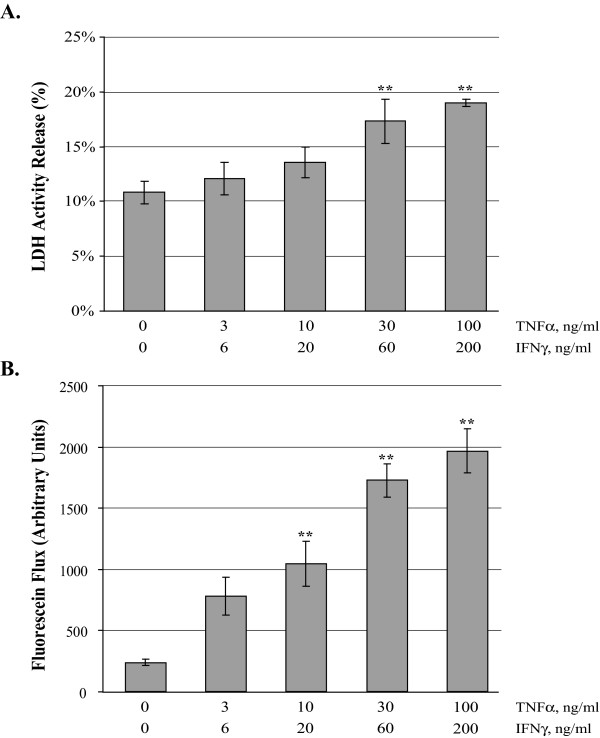
**Effect of Inflammatory Cytokine Concentration on MDCK Cell Barrier Function**. Lactate dehydrogenase release (Panel A) was measured in confluent MDCK cell cultures 24 hours following exposure to increasing doses of TNFα and IFNγ. Results are expressed as percent of maximal LDH release determined by incubating MDCK cells with TX-100 (2%) five minutes prior to LDH activity assay. The mean LDH is reported, error bars represent the SE, four independent experiments were assayed in duplicate. Fluorescein flux (Panel B) was measured following 24 hour treatment with increasing dose of TNFα and IFNγ. Fluorescein (50 μM) was added to the apical chamber and recovery was measured from the basal chamber after a 120 minute incubation. The mean fluorescence is reported, error bars represent the SE of four independent experiments. A one-way analysis of variance (ANOVA) was performed, multiple comparisons between control and treatments were determined with the Bonferroni post test. **Indicates statistical difference (P < 0.001) to control.

In order to determine whether TNFα/IFNγ induced cytotoxic effects in the MDCK cell cultures, we determined the percentage of apoptotic cells in confluent MDCK cultures using the TUNEL assay (Table 1) and measured LDH enzyme activity (Figure 1A) released from treated confluent cultures. No significant differences were found in percent of TUNEL positive cells following treatment for 24 hours with increasing doses of TNFα/IFNγ. As a positive control, cells were serum and glucose starved for 24 hours, and this resulted in a significant increase in TUNEL positive cells (Table 1). The LDH activity assay revealed that exposing MDCK cell cultures to increasing doses of TNFα/IFNγ for 24 hours produced an increase in LDH activity (Figure 1A) that was significantly different from media alone controls at the two highest doses 6.5* ± 2.0%, and 8.2* ± 0.3%. Examination of MDCK cultures by phase-contrast microscopy indicates that even at the highest experimental doses of TNFα/IFNγ, cell monolayers are intact. Using an established model of apoptosis and necrosis [27], MDCK cells were serum and glucose starved for 21 hours followed by addition of antimycin A (10 μM) for the final 3 hours to deplete ATP, this resulted in release of LDH that was significantly greater than the highest TNFα/IFNγ treatments (data not shown).

**Table 1:** Determination of Apoptosis in MDCK Monolayers. Confluent MDCK cultures were placed in one of the indicated treatment groups for 24 hours. Cells were then fixed and apoptotic cells were label using the TUNEL technique. Fluorescent microscopy was used to analysze of total cell number by DAPI staining and apoptotic cells by fluorescein incorporation. The mean ± SE of TUNEL positive cells is reported from three independent experiments. ANOVA was performed followed by a Bonferroni post test with all comparisons against the cells in media containing 5% FBS and glucose, *P < 0.01.

Glucose	+	+	+	+	-
Serum, 5%	+	+	+	+	-
IFNγ, ng/ml	-	20	60	200	-
TNFα, ng/ml	-	10	30	100	-
TUNEL Positive (%)	4.1 ± 0.7	4.3 ± 0.9	5.5 ± 1.3	5.9 ± 1.3	10.5 ± 1.3*

Depriving MDCK cells of serum and glucose for 24 hours resulted in a significant increase in paracellular flux with a corresponding decrease in transepithelial electrical resistance (Table 2), when deprived cells were depleted of ATP, flux increased dramatically while TER values declined to levels near baseline. Interestingly, when serum and glucose deprived cells were treated with TNFα/IFNγ (30/60 ng/ml) the TER values reversed and increased compared to starvation alone. Collectively, these data support the hypothesis that the combination of these cytokines alters tight junction function independently from apoptotic or necrotic mechanisms.

**Table 2:** Effect of Media Composition on MDCK Barrier Function. Confluent MDCK cultures were placed in one of the indicated treatment groups for 24 hours. Antimycin A (10 μM) was added at 21 hours following onset of experiment. The mean TER and fluorescein flux ± SE is reported from four independent experiments. ANOVA was performed followed by a Bonferroni post test with all comparisons against the cells in media containing 5% FBS and glucose, *P < 0.01.

Glucose	+	-	-	+	-
Serum, 5%	+	-	-	+	-
IFNγ, ng/ml	-	-	-	60	60
TNFα, ng/ml	-	-	-	30	30
Antimycin A, 10 μM	-	-	+	-	-
					
TER, Ω*cm^2^	72.3 ± 0.5	62.2 ± 2.5	7.1 ± 0.3*	162.2 ± 3.5*	105.0 ± 4.9*
Fluorescein Flux, AU	247.9 ± 8.5	1198.2 ± 52.1*	9201.7 ± 250.5*	2005.3 ± 50.5*	2440.3 ± 45.6*

### Effect of TNFα and IFNγ on transepithelial electrical resistance

**Figure 2:**
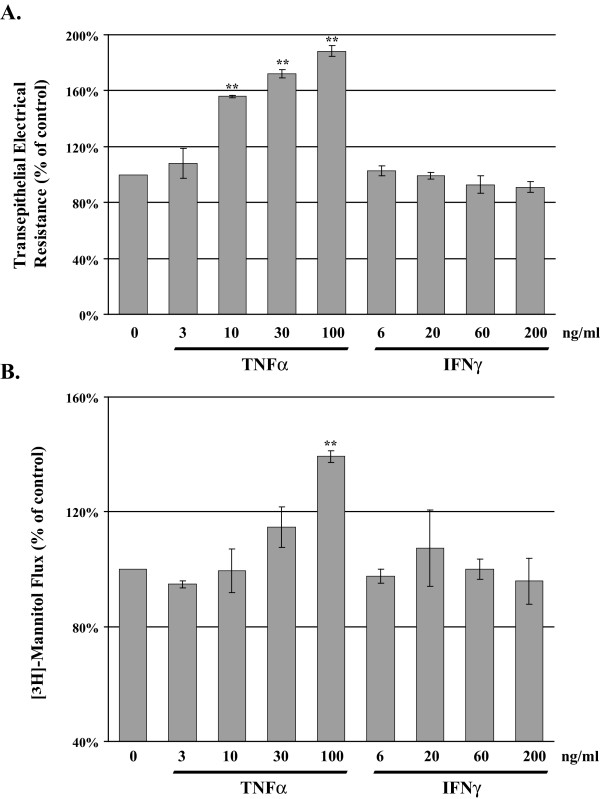
**Tumor Necrosis Factor-α Elevates Transepithelial Electrical Resistance and Flux in MDCK cells**. MDCK cells were treated with increasing dose of TNFα or IFNγ for 24 hours; TER and [^3^H]-mannitol flux were determined. Panel A reports the mean percent change in TER, TNFα produced a significant dose dependent elevation in TER whereas IFNγ had minimal effects on TER. At 24 hours post treatment [^3^H]-mannitol (2 μCi) was added to the apical chamber, cells were incubated at 37°C for two hours, recovery of tracer was measured in the basolateral chamber and expressed as fold change from the control group. Error bars represent the SEM, n = 3. A one-way analysis of variance (ANOVA) was performed, multiple comparisons between control and treatments were determined with the Bonferroni post test. **Indicates statistical difference (P < 0.001) to TNFα group (3 ng/ml).

Analysis of transepithelial electrical resistance (TER) in MDCK cells provides a reliable method for estimation of tight junction integrity. Confluent MDCK cell cultures were exposed to either TNFα or IFNγ for 24 hours before TER was measured. Exposure to TNFα induced a dose dependent elevation in TER (Figure 2A), whereas exposure to IFNγ induced no significant effect on MDCK cell TER.

**Figure 3:**
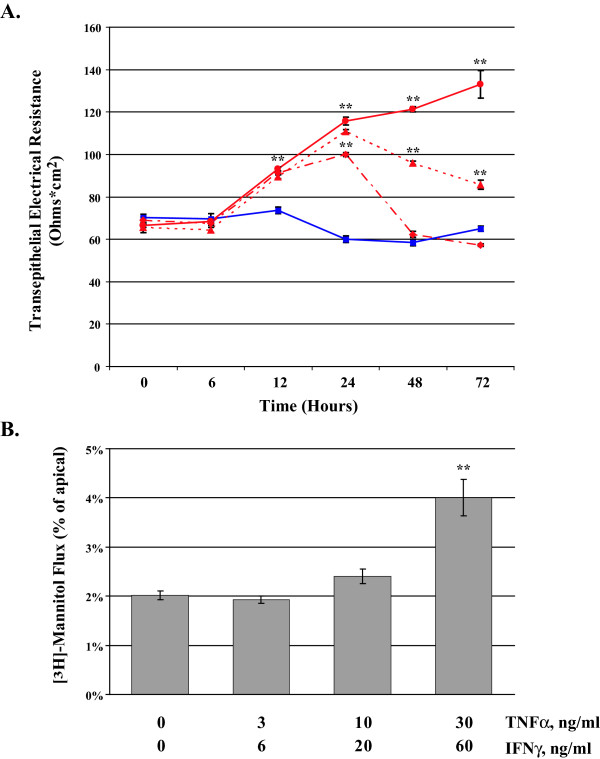
**Tumor Necrosis Factor-α and Interferon-γ Synergize to Elevate Flux in MDCK cells**. MDCK cells were treated with increasing dose of TNFα/IFNγ for up to 72 hours; TER and [^3^H]-mannitol flux were determined. Panel A reports the mean TER when cells were exposed to the following conditions; media only control (), TNFα/IFNγ, 3/6 ng/ml (), TNFα/IFNγ, 10/20 ng/ml (), and TNFα/IFNγ, 30/60 ng/ml (). Panel B reports the mean [^3^H]-mannitol flux following 72 hour incubation with the indicated treatments. Flux is presented as the percent of apical [^3^H]-mannitol recovered in the basolateral chamber following 120 min. incubation. Error bars represent the SE, n = 6. A one-way analysis of variance (ANOVA) was performed, multiple comparisons between control and treatments were determined with the Bonferroni post test. **Indicates statistical difference (P < 0.001) to control.

A time-course was performed for up to 72 hours on confluent MDCK cell cultures exposed to TNFα/IFNγ while TER was monitored at regular intervals. Figure 3A represents the changes in TER under the following conditions media only control, TNFα/IFNγ, 3/6 ng/ml, TNFα/IFNγ, 10/20 ng/ml, and TNFα/IFNγ, 30/60 ng/ml. In the first six hours of cytokine exposure TER values are relatively stable. Between 12 and 24 hours, a significant dose-dependent elevation of TER is observed. MDCK cells have 70.3 ± 0.6 Ω*cm^2 ^of resistance, at 24 hours cells treated with 3/6 ng/ml of TNFα/IFNγ developed 99.9 ± 0.8 Ω*cm^2 ^and 115.7 ± 1.9 Ω*cm^2 ^when treated with 30/60 ng/ml of TNFα/IFNγ. This represents a 65% increase in TER at 24 hr in the presence of the highest concentration of cytokine. Interestingly, between 24 and 72 hours there is a return toward baseline in MDCK cells treated with the lower doses of cytokine, whereas cells treated with the highest dose show a 104% increase in TER. These studies imply that treatment with TNFα/IFNγ in MDCK cells positively regulates factors that contribute to TER.

**Figure 4:**
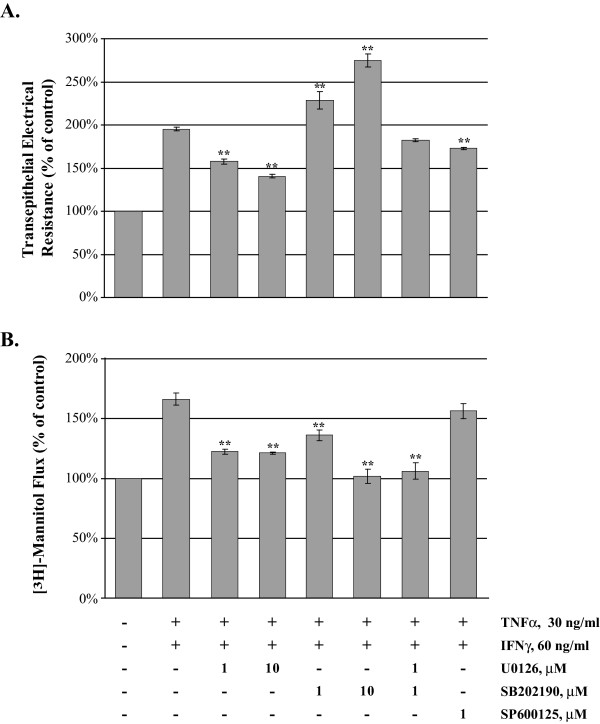
**Effect of MAP Kinase Inhibition on TER and Paracellular Flux in TNFα and IFNγ-treated MDCK cells**. The effect of the MAP kinase inhibitors was investigated by TER assessment and [^3^H]-mannitol flux determination in the presence of TNFα and IFNγ. MDCK cells were placed into one of eight treatment groups for 24 hours; control, TNFα/IFNγ alone (30/60 ng/ml) or proinflammatory cytokine with U0126 (1 and 10 μM), SB202190 (1 and 10 μM), combined U0126 and SB202190 (1 μM each) or SB600125 (1 μM). TER was assessed using the EVOM system (Panel A) then flux was determined following incubation at 37°C for two hours with [^3^H]-mannitol in the apical chamber (Panel B). Recovery of tracer was measured in the basolateral chamber and expressed as fold change from the control group. Exposure to TNFα/IFNγ produces a significant two-fold elevation in paracellular flux; MAP kinase inhibitors protect barrier function to varying degrees. Error bars represent the mean ± SE of four independent experiments. ANOVA was performed, multiple comparisons between all treatments were determined with the Tukey-HSD post test. **Indicates statistically difference (P < 0.01) to the TNFα/IFNγ group.

To investigate the contribution of the MAP kinase signaling pathway we employed several potent and specific pharmacological agents. MDCK cell grown to confluence on Transwell inserts were treated with TNFα/IFNγ (30/60 ng/ml) for 24 hr in the presence and absence of a panel of inhibitors (Figure 4A), U0126 (1 and 10 μM), SB202190 (1 and 10 μM) and a SP600125 (1 μM). Treatment with TNFα/IFNγ resulted in a 95% increase in TER compared to control cells, the addition of U0126 to cells treated with cytokine resulted in a significant dose dependent decrease in TER (37% and 55%). However, the treatment with SB202190, a p38 inhibitor produced a significant elevation of TER compared to cytokine alone, resulting in a dose dependent increase of 33% and 80%. The combination of low doses of ERK1/2 and p38 inhibition in the presence of cytokine produced minimal effect on the cytokine-treated cells, due to their opposing action on TER. The addition of SP600125, a JNK inhibitor did lower TER values a modest 22% in the presence of cytokine. MAP kinase activation and signaling pathways differentially regulate TER in this model of cytokine exposure in MDCK cells.

### Proinflammatory cytokines elevate flux

The effect of flux assay temperature in confluent MDCK cell cultures was determined, cells were placed into one of two treatment groups for 24 hours; control or TNFα/IFNγ (10 and 20 ng/ml). The paracellular flux tracer 4-kD FITC-dextran (30 μM) was added to the apical chamber and recovery was determined from the basolateral chamber at given intervals. We observed a modest six percent increase when the flux assay was performed at 37°C compared to 4°C using FITC-dextran. Additionally, exposure to TNFα/IFNγ did not markedly alter transcellular permeability. We observed an eight percent increase in FITC-dextran flux at 37°C when compared to the 4°C group. The modest increase in FITC-dextran recovery due to elevated temperature was expected, however, was not significantly different from the 4°C treatment (data not shown).

In order to examine the effect of dose of TNFα/IFNγ on epithelial barrier function confluent MDCK cultures were treated for 24 hours then fluorescein flux was determined. Fluorescein recovery was markedly elevated with increasing dose of TNFα/IFNγ (Figure 1B). The lowest dose tested resulted in a two-fold elevation in flux, doses of TNFα/IFNγ (10/20, 30/60 and 100/200 ng/ml) produced a dose dependent significant elevation in flux.

In Figure 2B, we report the [^3^H]-mannitol flux using MDCK cultures treated for 24 hours with either TNFα or IFNγ. [^3^H]-mannitol (2 μCi) was added to the apical chamber and recovery was measured in the basolateral chamber following a 120 minute incubation at 37°C. By in large MDCK cell flux is resistant to effects of these cytokines when administered alone, only the highest dose of TNFα (100 ng/ml) produced a significant 40% elevation of paracellular flux compared to control. IFNγ alone had no detectable effect on MDCK flux.

In Figure 3B, we report the [^3^H]-mannitol flux using MDCK cultures treated for 72 hours with TNFα/IFNγ. In control cells, approximately 2% of the [^3^H]-mannitol is recovered in the basolateral chamber following a 120 minute assay. MDCK cells treated with TNFα/IFNγ (30/60 ng/ml) produce a two-fold increase in [^3^H]-mannitol flux permitting 4% of the label to enter the basolateral compartment. In this model of inflammatory stress using MDCK cells, TER and flux appear not to be inversely related.

We investigated the effect of MAP kinase pathway inhibitors on paracellular flux using MDCK cells exposed to TNFα/IFNγ (30/60 ng/ml) for 24 hr. Following analysis of TER, [^3^H]-mannitol flux assays were performed and reported as percent of control (Figure 4B). TNFα/IFNγ resulted in a 66% increase in flux compared to control cells, U0126 significantly decreased flux (44 and 45%), SB202190 decreased flux dose-dependently (30% and 64%), the combination of U0126 and SB202190 significantly lowered flux 60% back to control levels whereas SP600125 did not significantly alter flux. In these experiments, inhibition of ERK1/2 and/or p38 signaling during TNFα/IFNγ exposure significantly protects MDCK cell barrier function.

### Western blot analysis of tight junction- related proteins

**Figure 5:**
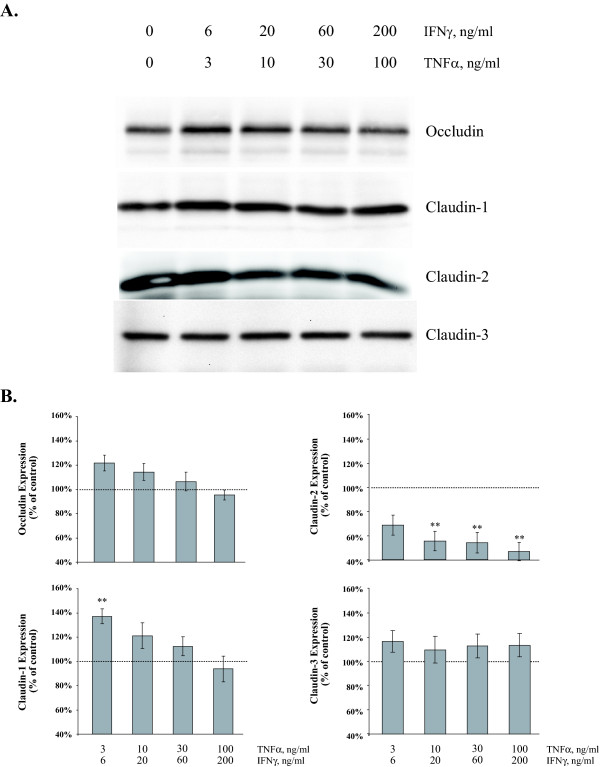
**Effect of Proinflammatory Cytokine Concentration on MDCK Cell Tight Junction Proteins**. Representative immunoblots of occludin, claudin-1, claudin-2 and claudin-3 total protein from confluent MDCK cell cultures twenty-four hours following exposure to increasing doses of TNFα and IFNγ (Panel A). Immunoblots were subjected to densitometric analysis; results are reported as percent of control to examine the effect of proinflammatory cytokines on tight junction protein expression (Panel B). Dashed horizontal lines represent control level of expression (100%), error bars represent the mean ± SE of eight independent experiments. ANOVA was performed, multiple comparisons between control and treatments were determined with the Bonferroni post test. **Indicates statistically difference (P < 0.01) to control.

To examine the causative factors related to elevated paracellular flux and decreased TER, selected tight junction gene products were examined by Western Blot. Occludin, claudin-1, claudin-2 and claudin-3 expression were examined in MDCK cells in response to TNFα/IFNγ dose following a twenty-four hour exposure (Figure 5A). In general, low dose of TNFα/IFNγ produces an initial elevation in tight junction protein expression with the exception of claudin-2; the intensity and patterns observed vary. In Figure 5B, exposure to TNFα/IFNγ (3/6 ng/ml) for twenty-four hours induced a significant 37% ± 6% elevation in claudin-1 expression when compared to control as measured by densitometric analysis. The occludin expression pattern (Figure 5B) is comparable to claudin-1, however the magnitude of elevation is modest, only a 22% ± 6.5% was observed at the lowest TNFα/IFNγ dose. Claudin-2 levels decrease markedly in a dose-dependent manner to 45% ± 7% of control values. Analysis of claudin-3 (Figure 5B) indicated a slightly different expression pattern; we observed a modest but not significant increase with all doses of TNFα/IFNγ tested.

**Figure 6:**
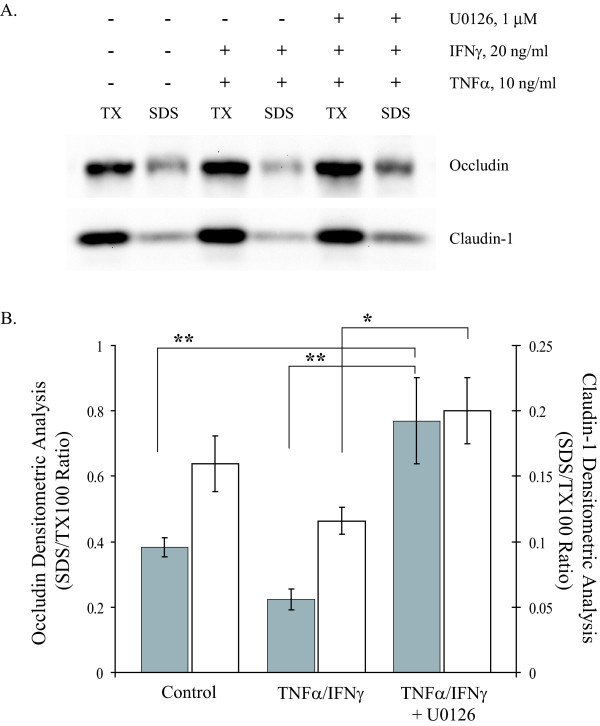
**ERK1/2 Inhibition Reverses Cytokine-induced Alterations in Tight Junction Protein Distribution**. Representative immunoblots of occludin and claudin-1 using Triton X-100 soluble and insoluble (SDS) fractions from confluent MDCK cell cultures treated for 24 hours in the indicated conditions (Panel A). The effect of the MEK inhibitor (U0126, 1 μM) was added fifteen minute prior to addition of proinflammatory cytokines. Densitometic analyses were performed, occludin (filled bars) and claudin-1 (open bars), ratio of SDS to TX-100 intensity was reported. Error bars represent the mean ± SE of four independent experiments. ANOVA was performed, multiple comparisons between all treatments were determined with the Tukey-HSD post test. **Indicates statistically difference (P < 0.05) to the control group, ***indicates a significant difference to the TNFα/IFNγ group (P < 0.05).

In order to examine dynamic changes in protein expression a differential detergent extraction procedure was employed using cytokine-treated MDCK cells. Representative immunoblots of occludin and claudin-1 are presented in Figure 6A. The densitometric ratio of TX100-insoluble fraction to TX100-soluble fraction for occludin and claudin-1 was reported in Figure 6B. TNFα/IFNγ (10/20 ng/ml) exposure for 24 hours resulted in decreased occludin and claudin-1 in the TX100-insoluble fraction. MDCK cells pretreated with U0126 (1 μM) for 15 minutes prior to addition of TNFα/IFNγ showed a significant elevation of occludin and claudin-1 in the TX100-insoluble fraction, this finding is consistent with the improved barrier function in ERK inhibited-TNFα/IFNγ-treated MDCK cells. These data suggest that early MAP kinase signaling events following TNFα/IFNγ exposure lead to both physical and functional remodeling of key tight junction proteins.

### Tight junction localization studies

**Figure 7:**
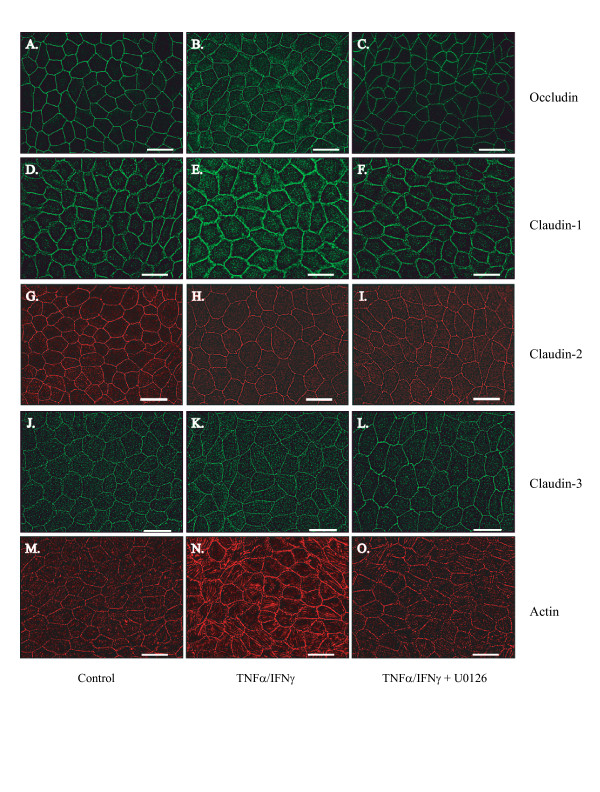
**Aberrant Localization of Tight Junction Proteins and Cytoskeletal Reorganization induced by Proinflammatory Cytokines in MDCK Cells**. Immunofluorescence microscopy was used to capture digital images of MDCK cells grown to confluency on glass coverslips. Images A, D, G, J and M show control occludin, claudin-1, claudin-2, claudin-3 and actin staining respectively. Images B, E, H, K and N demonstrate occludin, claudin-1, claudin-2, claudin-3 and actin staining following twenty-four treatment with TNFα/IFNγ (10 and 20 ng/ml). Cells were treated TNFα/IFNγ for a 24 hour interval in the presence the ERK1/2 inhibitor U0126, 1 μM, representative images C, F, I, L and O were stained for occludin, claudin-1, claudin-2, claudin-3 and actin respectively. All images were captured on a Nikon 2000E microscope using a 60X oil-immersion lens, the calibration bar represents 25 μm.

Occludin, claudin-1, claudin-2 and claudin-3 localization was examined using indirect immunofluorescence in MDCK cells cultured on coverslips. Cells were treated for 24 hours in the absence and presence of TNFα/IFNγ. In this study, differences in expression and localization were quantified by examining junctional fluorescent intensity in a deconvoluted z-stack comparing the highest intensity of staining to the adjacent intracellular location (J/I ratio). Occludin expression is robust and localized discreetly to the cell's periphery (Figure 7A). Examination of the effect of TNFα/IFNγ dose demonstrates an elevated occludin signal with a substantial increase detected at the cell-cell contact regions (Figure 7B). Analysis of occludin fluorescence intensity at the junction demonstrates a significant 55% increase in signal detected. Claudin-1 staining is more dynamic than occludin but generally the signal is localized to the periphery (Figure 7D). TNFα/IFNγ treatment produces a marked elevation of claudin-1 cytoplasmic staining; the addition of U0126 restores claudin-1 localization to the periphery (Figure 7E). Claudin-2 staining is localized to the periphery (Figure 7G), the TNFα/IFNγ exposure produced a significant decrease in junctional intensity (Figure 7H), U0126 produced little restoration of claudin-2 staining in the presence of TNFα/IFNγ. In contrast, claudin-3 staining (Figure 7J-L) and J/I analysis demonstrated that exposure to TNFα/IFNγ has minimal effect on claudin-3 localization.

### Actin stress fiber analysis

Actin staining was examined using Texas-Red phallodin in MDCK cells following twenty-four hour exposure to the given treatments (Figure 7J-L). In this study, perijunctional actin staining was analyzed in z-sections that corresponded to the apical region containing the most intense staining of a tight junction marker. Perijunctional actin staining increased by more than a factor of two in the TNFα/IFNγ-treated group (Table 3). Actin staining was similar to the control group when TNFα/IFNγ was delivered in the presence of U0126.

**Table 3:** Fluorescence Analyses of Junction Proteins and the Actin Cytoskeleton. Fluorescence intensity measurements were made using the line tool in MetaMorph Image Analysis software at the cell junction. The ratio of junctional to intracellular fluorescent is reported and the fluorescent intensity at the cell junctions are reported ± SEM. Actin staining was quantified at apical z-section stacks that corresponded to the same plane as the tight junction. Fluorescent intensity (arbitrary units) at the cell junction is reported in the lower panel. ANOVA was performed followed by a Bonferroni post test with all comparisons against the control group. A minimum of 50 junctions were analyzed in each group *P < 0.001.

	**Fluorescence Intensity Ratio (Junctional/Intracellular)**		
Tight Junction Protein	Control	TNFα/IFNγ (10 and 20 ng/ml)	TNFα/IFNγ with U0126 (1 μM)

Occludin	29.12 ± 1.79	*16.24 ± 0.78	*21.42 ± 1.15
Claudin-1	29.70 ± 2.79	*11.65 ± 1.53	*22.08 ± 1.51
Claudin-2	29.97 ± 1.76	*24.57 ± 1.98	*21.35 ± 1.73
Claudin-3	7.27 ± 0.57	7.11 ± 0.71	9.45 ± 0.88
			
	**Fluorescence Intensity (Junctional)**		
			
Actin	91.55 ± 5.2	*191.34 ± 10.6	116.5 ± 5.0
Occludin	644.7 ± 25.8	*917.6 ± 28.4	*1238.1 ± 30.2
Claudin-1	668.3 ± 27.6	*469.3 ± 22.9	557.4 ± 21.7
Claudin-2	545.4 ± 24.3	*364.0 ± 12.7	*357.4 ± 19.3
Claudin-3	204.8 ± 18.2	212.7 ± 18.3	250.0 ± 18.7

## Discussion

Epithelial cell layers play a critical role by separating physiologically distinct compartments within most major organ systems. The loss of tight junction barrier function by deleterious inflammatory mechanisms is an important problem in renal physiology due to the contribution of these structures to the maintenance of ionic and water balance. Acute renal failure related to ischemic episodes profoundly impairs the renal epithelium. Following ischemia-reperfusion injury, cytoskeletal disruption and loss of epithelial cell polarity contribute to back-leak of glomerular filtrate into the blood [28]. In a recent study following kidney transplant, decreased ZO-1 staining was reported in response to postischemic injury [29]. Importantly, leukocyte infiltration is likely to occur in chronic renal conditions such as diabetes, hypertension, autoimmune disorders leading to production of proinflammatory cytokines [30-32]. In this study, we employ an important renal cell model, (MDCK cells) to examine the effects of proinflammatory cytokines on barrier function.

Initially, we were interested in determining whether the proinflammatory treatment regiment was altering MDCK barrier function as a cytotoxic effect. TNFα is a known activator of NF-κB, a transcription factor that promotes survival; in a recent report NF-κB activity was inhibited by exogenous expression of Smad7 resulting in elevated apoptosis in MDCK cells [33]. However, it was recently reported that TNFα (30 ng/ml) initiated caspase-8 cleaved PARP that induced apoptosis in serum starved MDCK cells [34]. In the present study, MDCK cells were treated in media containing five percent FBS to minimize serum withdrawal responses, we report that the combination of cytokines used in this study did not significantly induce apoptosis. At the highest doses (30/60 and 100/200 ng/ml) of cytokine treatment there was a moderate elevation in LDH release, however this was less than a ten percent elevation in LDH levels compared to control. Importantly, we report that paracellular flux increased in a graded fashion with increasing dose of TNFα/IFNγ. When renal epithelial cells are exposed to agents that produce necrosis and apoptosis investigators report a decrease in TER along with a subsequent increase in paracellular flux [27,35], we confirmed this finding in the MDCK system by using a combination of energy starvation and ATP depletion. We find that exposure of MDCK cells to TNFα/IFNγ results in a decrease in ionic permeability which is reported as increased TER values, in fact when MDCK cells are serum and energy starved ionic permeability decreased in response to TNFα/IFNγ. These data suggest that the MDCK cell response to TNFα/IFNγ is distinct from a cytotoxic insult. In support of this concept a recent study using the intestinal epithelial T84 cell line demonstrated that the combination of TNFα/IFNγ increases paracellular permeability in an apoptosis-independent manner [36]. Therefore, although it is feasible to induce cell death in MDCK cells by serum starvation and/or high doses of TNFα for an extended duration, we are confident that the perturbations reported in barrier function were conducted using conditions that would activate NF-κB minimizing induction of apoptotic events. These conditions appear to result in a reorganization of the MDCK cell junctions with minimal loss of junctional proteins.

In the present study we have demonstrated that pharmacological inhibition of MEK1 and p38 signaling in proinflammatory cytokine stimulated MDCK cells functionally protects the barrier function. Several studies indicate that MEK1 signaling increases paracellular permeability, there exists some disparity in observed cellular responses. Recently, a report demonstrated that inhibition of MEK1 signaling did not influence expression of occludin or claudin-1 or affect tight junction function in several breast cancer cell lines [37]. Also, a study using enteropathogenic *Escherichia coli*, showed that ERK1/2 was activated in T84 cells, but did induce tight junction barrier disruption as measured by TER [38]. However, activation of MEK1 signaling by H_2_O_2 _exposure in endothelial cells increased permeability and resulted in occludin disorganization [39]. Similar effects were also observed in Caco-1 and MDCK cell lines [40]. In this present study, activation of the ERK1/2 pathway by TNFα/IFNγ treatment produced altered ionic permeability and dynamic changes in junctional protein expression and localization. Additionally, we found that TNFα alone potently decreased MDCK cell ionic permeability while having only minimal impact on paracellular flux. This suggests that the observed junctional responses occur independent of apoptotic or necrotic mechanisms that likely elevate paracellular flux.

Decreased ionic permeability in response to TNFα or TNFα/IFNγ exposure coupled to the increased paracellular flux of non-charged solutes when cytokines were presented in combination is intriguing. We find that inhibition of ERK1/2 signaling increased ionic permeability toward control levels as expected but inhibition of p38 signaling further decreased ionic permeability levels above cytokine treatment alone. This suggests that activation of the p38 pathway is antagonizing ERK1/2-mediated effects on elevated TER in TNFα/IFNγ-treated MDCK cells. While the MAP kinase inhibitors produced divergent effects on cellular ionic permeability measurements both inhibitors protected against increase paracellular flux of non-charged solutes. Several recent reports reveal that ERK1/2 activation in MDCK II cells results in increased TER. For instance, a recent study of cyclosporine A treated MDCK cells produced elevated TER through a MAPK pathway [41]. In another study of MDCK II cells, EGF receptor activation resulted in increased TER with a concomitant decrease in claudin-2 expression [7]. In a recent study of MDCK II cells investigators demonstrate that these cells have endogenously low ERK1/2 activity that corresponds to high expression of claudin-2 [42]. ERK1/2 inhibition in all of these studies prevented elevation of TER in the MDCK II cell line. Recently investigators have determined that claudin-2 forms cation-selective channels in the tight junction complex [43,44], alteration in claudin-2 expression results in perturbations in ionic permeability. Consistent with these studies we find a dose-dependent decrease in claudin-2 expression in MDCK cells treated with TNFα/IFNγ, this loss of claudin-2 correlates to a substantial reduction in ionic permeability. Elevation in TER was inhibited by treatment with the ERK1/2 inhibitor but not by inhibiting the p38 signaling pathway. These findings are consistent with the current literature demonstrating that claudin-2 levels are regulated following ERK1/2 activation in MDCK cells and its expression level will influence recorded TER from MDCK cultures.

The cellular tight junction response to proinflammatory cytokines is variable based on cell type and numerous physiological variables. Measurable changes in tight junction protein expression or localization that are predicted to play a key role in maintaining barrier function are typically more unpredictable. We report a statistically significant elevation in the protein expression of claudin-1, but not occludin or claudin-3, following exposure to TNFα/IFNγ. However, occludin protein levels are slightly elevated in response to several doses of TNFα/IFNγ tested compared to control. In this study, we report a dose-dependent decrease in claudin-2 expression following exposure to TNFα/IFNγ. The heterogeneous response of tight junctional proteins to cytokine exposure may be due to junctional remodeling which may involve additional protein synthesis and altered turnover rates. In other studies, researchers have reported decreases, increases or no change in tight protein expression following challenge with proinflammatory mediator. For instance: TNFα increased permeability while decreasing ZO-1 expression through increased NFκB signaling, in a study using Caco-2 cells [45]. Investigators report increased paracellular flux with a decrease in TER following TNFα/IFNγ exposure using a mouse cholangiocyte model; interestingly major structural changes to the tight junction proteins (occludin, claudin-1, -3, and ZO-1) were not detected [46]. Finally, using T84 cells investigators find that inhibition of MEK signaling impairs both basal and cytokine-induced tight junction formation demonstrating an increased claudin-1 and claudin-2 protein expression in response to the cytokine IL-17 [47]. Although it might be tractable to predict that exposure to proinflammatory cytokines would be correlated to decreased expression of tight junction proteins, our study is in agreement with other studies, finding moderate effects on expression.

In the present study's examination of tight junction protein localization, treatment with ERK1/2 inhibitor in the presence of TNFα/IFNγ enhanced occludin and claudin-1 expression at the junctional interface but did not significantly affect claudin-2 or claudin-3. In the TNFα/IFNγ treatment group there appears to be increased cytoplasmic staining, possibly related to a lack of tethering related to cytoskeletal rearrangements. Functionally, MDCK cells pretreated with the ERK1/2 inhibitor exhibited no change in flux or TER compared to control cells even in the presence of TNFα/IFNγ. Analysis of tight junction protein levels demonstrated that pretreatment with U0126 in the presence of TNFα/IFNγ induces protein levels similar to control however, when the ERK1/2 inhibitor was added two hours following treatment with TNFα/IFNγ, we observed a similar magnitude of elevation in their expression, similar to TNFα/IFNγ alone. This suggests that early events in the cytokine response are activated and produce lasting effects on MDCK cells.

The actin cytoskeleton maintains an intimate association with tight junctions through scaffolding proteins like ZO-1, a member of the MAGUK family [48,49]. A recent study using a glomerular epithelial cell model exposed to TNFα for twenty-four hours reports an approximate two-fold elevation in total actin content as determined by a DNase I inhibition assay [50]. This finding is consistent with our results based on perijunctional actin staining in MDCK cells. We report a two-fold elevation of F-actin staining following exposure to TNFα/IFNγ. This increase was prevented by inhibition of ERK1/2 activation. It is also noteworthy that other researchers have identified functional relationship between MEK activation and cytoskeletal organization. MEK-dependent pathway leads to cytoskeleton disruption in Ras-transformed fibroblasts [51]. Additionally studies examining epithelial dedifferentiation show a loss of actin stress fibers [24,52]. MDCK cells stimulated with HGF/SF produced actin cytoskeleton reorganization through the activation of MAP kinase, resulting in junctional rearrangement [53]. Constitutively active Raf-1 in a salivary epithelial cell model induced actin reorganization and disrupted tight junctions by downregulation of occludin [54]. The association between the cytoskeleton and the tight junction suggests a structural and functional relationship that provides a tractable model for understanding the regulation of barrier function.

## Conclusion

We demonstrated that MDCK tight junctions are functionally reorganized in response to TNFα/IFNγ exposure through the activation of ERK1/2. We find the junction tightens by elevating the expression of occludin and claudin-1 in response to TNFα/IFNγ. Additionally, decreased ionic permeability arises primarily through a significant loss of claudin-2 expression due to ERK1/2 activation. Apoptotic and necrotic mechanisms in response to TNFα/IFNγ may contribute in part to the elevated paracellular flux. Based on immunofluorescent findings, occludin and claudin-1 localization appear to be in transition perhaps due to the reorganization of the actin cytoskeleton. Activated ERK1/2 and p38 signaling pathways appear to be responsible in part for both the curative and disruptive patterns observed in this study.

## Methods

### Materials

Minimum Essential Medium Eagle (Mediatech, Herndon, VA), L-glutamine, sodium pyruvate, non-essential amino acids, fetal bovine serum (FBS), penicillin (200 U/mL), streptomycin (200 μg/mL), trypsin/(0.03%) solution and Transwell Systems were purchased from Fisher Scientific. Human recombinant TNFα was purchased from Becton-Dickinson (San Jose, CA). Human recombinant IFNγ was purchased from R&D Systems (Minneapolis, MN). U0126, SB202190 and SP600125 inhibitors were purchased from EMD Biosciences (San Diego, CA). Polyclonal rabbit anti-occludin, anti-claudin-1, anti-claudin-3 and monoclonal mouse anti-claudin-2 and antibodies were purchased from Zymed Laboratories (South San Francisco, CA). Horseradish Peroxidase (HRP) anti-rabbit IgG, HRP-anti-mouse IgG2b and Texas Red anti-mouse IgG2b antibodies were purchased from Jackson ImmunoResearch Laboratories, Inc. (West Grove, PA). Alexa488 anti-rabbit IgG antibody and Texas Red Phallodin were purchased from Molecular Probes (Eugene, OR). A cytotoxicity kit was supplied by Roche Applied Science (Indianapolis, IN). D-[2-^3^H]mannitol was purchased from Perkin Elmer (Wellesley, MA) and 4 KDa FITC-dextran from Sigma Chemical (St. Louis, MO). All other reagents were of the highest quality available.

### Cell culture

MDCK cells (CCL-34) were obtained from ATCC (Manassas, VA). MDCK cells were grown in Minimum Essential Medium Eagle supplemented with L-glutamine (2 mM), sodium pyruvate (1 mM), non-essential amino acids (0.1 mM), 5% FBS, penicillin (200 U/mL), streptomycin (200 μg/mL) in a humidified incubator at 37°C and 5% CO_2_. MDCK cells are passaged using a trypsin (0.25%), EDTA (0.03%) solution and culture dishes are reseeded following a 1:4 dilution. Laboratory grade water (Millipore) is used for all solutions and the water is routinely tested for the presence of endotoxin using the Limulus Amebocyte Lysate Assay (Sigma, St. Louis, MO).

### Cytotoxicity measurement

Lactate dehydrogenase (LDH) activity released into the supernatant of MDCK cell cultures was used as a measure of cytotoxicity, manufacturer's instructions were followed. Briefly, MDCK cells were grown to confluency in 24-well plates then placed into one of the following five treatment groups: control, or media containing TNFα and IFNγ with the indicated concentrations; 3 and 6 ng/ml, 10 and 20 ng/ml, 30 and 60 ng/ml or 100 and 200 ng/ml respectively. MDCK cells were treated for 20 hours in complete DMEM media and then placed in DMEM media containing 0.5% FBS without phenol red for the remaining 4 hours prior to assay. Cell culture plates were centrifuged for 5 min at 1000 g and 100 μl of supernatant was transferred to an optically clear flat-bottom 96-well microtiter plate. LDH activity assay was initiated by addition of 100 μl substrate and absorbance was measured at 492 nm using a SpectraMax250 Platereader (Molecular Devices). DMEM containing 0.5% FBS without phenol red was used as assay medium to determine low control. Additionally, a group of cells was lysed with 2% Triton-X100 ten minutes prior to supernatant collection to determine total cellular LDH activity.

Apoptosis was detected using the DeadEnd™ Fluorometric TUNEL System (Promega, Madison, WI). MDCK cells grown to confluency on tissue culture treated coverglasses were placed in a variety of conditions for 24 hours. Cells were then fixed with paraformaldehyde (4%) in PBS for 25 minutes then permeabilized in PBS containg 0.2% Trition X-100 for 5 minutes. DNA fragments were labeled with fluorescein-UTP using a recombinant terminal deoxynucleotidyl transferase for 1 hour at 37°C. Following six wash steps in 2× SSC and PBS, nuclei were stained with DAPI. Slides were stored in the dark at 4°C prior to microscopic analysis using a Nikon 2000E microscope.

### Transepithelial electrical resistance

MDCK cells are seeded on Transwell inserts and grown to confluency. Experiments are preformed on cultures after a minimum of ten days culture. In all experiments, cytokines and inhibitors were delivered to both the apical and basolateral chambers. Measurements of transepithelial electrical resistance (TER) were made using an EVOM epithelial voltohmmeter with an EndOhm 12 mm measurement chamber calibrated daily using CaliCell™ (World Precision Instruments, Sarasota, FL). Transwell inserts are transferred to the measurement chamber containing media (2 ml); the apical electrode is positioned prior to obtaining measurement. Readings taken at time 0 hrs were obtained immediately following addition of drug treatments. The resistance of the epithelium was determined by passing a bipolar current across the epithelium and measuring the resultant voltage change. The resistance of the fluid and insert only between the voltage measuring electrodes was measured and subtracted from the total resistance. The transepithelial resistance was automatically determined using Ohm's law.

### Paracellular flux assay

MDCK cell monolayers in Transwell inserts were incubated under different experimental conditions in the presence of 0.2 *μ*Ci/ml of D-[2-^3^H]-mannitol (15 Ci/mmol) or sodium fluorescein (50 μM) in the apical well. At given times, apical (30 *μ*l) and basal (90 *μ*l) media was withdrawn and radioactivity was counted with a scintillation counter. The flux into the basal well was calculated as the percentage of total isotope administered into the basal well per hour per cm^2 ^of surface area. At 120 minutes following fluorescein addition, basal media (90 *μ*l) was placed in a Corning 96-well black assay plate and fluorescein was determined using a Typhoon Trio Plus (GE Healthcare, Piscataway, NJ).

### Western blot analysis

Western blot analyses are processed using the following protocol. Briefly, to prepare total cellular protein MDCK cells are washed with cold PBS and lysed in buffer containing 1% Triton X-100, 1% sodium deoxycholate, 0.1% SDS, 2 mM EDTA, 0.15 M NaCl, 0.01 M NaPO_4_, mini-Complete protease inhibitor (Roche Applied Science) with the following phosphatase inhibitors: 2 mM Na_3_VO_4 _and 10 mM NaF. DNA is sheared using a small gauge needle, and insoluble material is precipitated by centrifugation. Supernatants were collected and stored at -20°C for analyses.

The method to prepare Triton X-100-soluble and -insoluble fractions was adapted from Singh *et. al*. [7] with minor modifications. MDCK cells were scraped into lysis buffer (10 mM HEPES, pH 7.2, 1% Triton X-100, 100 mM NaCl, 2 mM EDTA and protease inhibitors) and incubated for 20 min. at 4°C. Following centrifugation, supernatants were collected and considered the TX-100 soluble fractions, the pellets were placed in lysis buffer containing 1% SDS and TX-100 insoluble proteins were released by three sonication pulses (40% duty cycle at output control level 4) using a Branson Sonifier 450. Insoluble material was removed by centrifugation, supernatants were collected and stored at -20°C for analyses.

Protein concentration was determined using the Pierce BCA (Rockford, IL) microtiter plate protocol using albumin as a reference standard. Lysates are denatured at 95°C for 5 min in Lammeli sample buffer, electrophoresed on 10% SDS-PAGE gels, and electroblotted to PVDF membrane for immunodetection. Tight junction specific antisera including anti-occludin and claudin-1 and -3 were employed in this study. Immunoblots are processed by blocking non-specific binding sites in 5% non-fat milk in Tris buffered saline with 0.1% Tween 20 (TBS-T) for 30 minutes followed by incubation with diluted primary antibody (1/5000) for 2 hours at room temperature. Immunoblots are then washed three times in TBS-T followed by incubation with an HRP-conjugated secondary antibody (1/10,000). Following extensive washing with TBS-T, immunoblots are developed with a stable West Pico chemiluminescent substrate (Pierce, Rockford, IL). The image was captured on the VersaDoc 3000 and analyzed with the integrated QuantityOne 1-D analysis software.

### Immunofluorescent analysis

MDCK cell monolayers were grown on culture-treated cover slips and treated for 24 hours in one of the following conditions: media only, TNFα/IFNγ (10/20 ng/ml), or TNFα/IFNγ with U0126 (1 μM, 15 min. pretreatment). Layers were rinsed once with sterile PBS and placed on ice for ten minutes. Cells were permeabilized with an actin stabilizing permeabilization buffer containing 0.2% Triton-X100, 100 mM KCl, 3 mM MgCl_2_, 1.3 mM CaCl_2_, 25 mM sucrose, and 2 mM HEPES, pH 7.1 for 2 min on ice. Cells were then fixed with cold 95% ethanol in PBS for 30 min on ice, rinsed once with PBS and blocked with 1% BSA in PBS for 10 min followed by incubation for 1 hour with tight junction protein-specific primary antibodies in a moist environment at 25°C. Cells were rinsed three times with PBS and incubated with Alexa488-conjugated antibodies for 45 min. Primary and secondary antibodies were diluted into 0.2% BSA in PBS and spun at 10,000 × g for 15 min at 4°C before incubation. Rhodamine-phalloidin (50 ng/ml) staining was performed after three PBS washes for 20 min. Following extensive rinse steps, coverslips are coated with anti-fade medium and stored in the dark at 4°C prior to microscopic analysis using a Nikon 2000E microscope fitted with a z-stepper motor and MetaMorph Image Analysis Software. Fluorescent intensity was measured from a minimum of 50 cell junctions per slide, data from a minimum of three independent experiments were pooled for analysis.

### Statistics

Multiple comparisons were made using one-way analysis of variance (ANOVA) followed by either the Bonferroni when comparing multiple samples to control or Tukey HSD post-hoc test. A p value < 0.05 was considered significant.

## Authors' contributions

Author DMP performed the cytotoxicity, immunofluorescent analysis and tight junction expression studies, authors AKL and JJS performed the paracellular flux and MAP kinase inhibition studies and author KAD participated in the TER studies. Author JMK oversaw and was involved all of the studies. All of the authors contributed to drafting the manuscript.

All authors read and approved the final manuscript.
